# NK and NKT Cell-Mediated Immune Surveillance against Hematological Malignancies

**DOI:** 10.3390/cancers12040817

**Published:** 2020-03-28

**Authors:** Kanako Shimizu, Tomonori Iyoda, Satoru Yamasaki, Norimitsu Kadowaki, Arinobu Tojo, Shin-ichiro Fujii

**Affiliations:** 1 Laboratory for Immunotherapy, RIKEN Center for Integrative Medical Sciences, 1-7-22, Suehiro-cho, Tsurumi-ku, Yokohama, Kanagawa 230-0045, Japan; tomonori.iyoda@riken.jp (T.I.); satoru.yamasaki@riken.jp (S.Y.); 2 Department of Internal Medicine, Hematology, Rheumatology and Respiratory Medicine, Faculty of Medicine, Kagawa University, 1750-1 Ikenobe, Miki-cho, Kita-gun, Kagawa 761-0793, Japan; kado@med.kagawa-u.ac.jp; 3 Department of Hematology/Oncology, The Institute of Medical Science, The University of Tokyo, Minato, Tokyo 108-8639, Japan; a-tojo@ims.u-tokyo.ac.jp

**Keywords:** innate immunity, NK cells, iNKT cells, dendritic cells, hematological malignancy

## Abstract

Recent cancer treatment modalities have been intensively focused on immunotherapy. The success of chimeric antigen receptor T cell therapy for treatment of refractory B cell acute lymphoblastic leukemia has pushed forward research on hematological malignancies. Among the effector types of innate lymphocytes, natural killer (NK) cells show great importance in immune surveillance against infectious and tumor diseases. Particularly, the role of NK cells has been argued in either elimination of target tumor cells or escape of tumor cells from immune surveillance. Therefore, an NK cell activation approach has been explored. Recent findings demonstrate that invariant natural killer T (iNKT) cells capable of producing IFN-γ when optimally activated can promptly trigger NK cells. Here, we review the role of NKT and/or NK cells and their interaction in anti-tumor responses by highlighting how innate immune cells recognize tumors, exert effector functions, and amplify adaptive immune responses. In addition, we discuss these innate lymphocytes in hematological disorders, particularly multiple myeloma and acute myeloid leukemia. The immune balance at different stages of both diseases is explored in light of disease progression. Various types of innate immunity-mediated therapeutic approaches, recent advances in clinical immunotherapies, and iNKT-mediated cancer immunotherapy as next-generation immunotherapy are then discussed.

## 1. Introduction

Cancer immunotherapy, which works by activating the immune system, has become an important treatment option for several cancers. Recently, successful clinical anti-tumor treatments with antibodies and cell therapy have become landmark events in the history of cancer therapy [1,2,3]. In fact, immune checkpoint blockade (ICB) with anti-programmed cell death 1 (anti-PD-1), anti-programmed cell death ligand 1 (anti-PD-L1), and anti-CTLA-4 antibodies have demonstrated their clinical efficacy in treating previously untreatable advanced-stage cancer patients since 2011 [1,2]. This discovery of the inhibition of negative immune regulation as a means of cancer therapy led to James P. Allison and Tasuku Honjo being awarded the Nobel Prize in Physiology or Medicine in 2018. As a cell-based immunotherapy, the US Food and Drug Administration (FDA) approved chimeric antigen receptor (CAR) T cell therapy for the treatment of refractory B cell acute lymphoblastic leukemia in 2017 [3]. These clinical successes are mainly due to the T cell-centered view of tumor immunity. However, T cells are not autonomous in their effector functions. The onset and maintenance of T cell responses and the development of protective memory T cells sometimes depend on innate immune responses. 

The innate immune system, as the first line of defense, is implicated in an enormous number of disease processes by detection of invaders such as pathogenic microorganisms (viruses, bacteria, and parasites) and tumors. Upon detection, the innate immune system activates cells to attack and destroy these microorganisms or initiate repair, while also informing and modulating the adaptive immune response.

**Figure 1:**
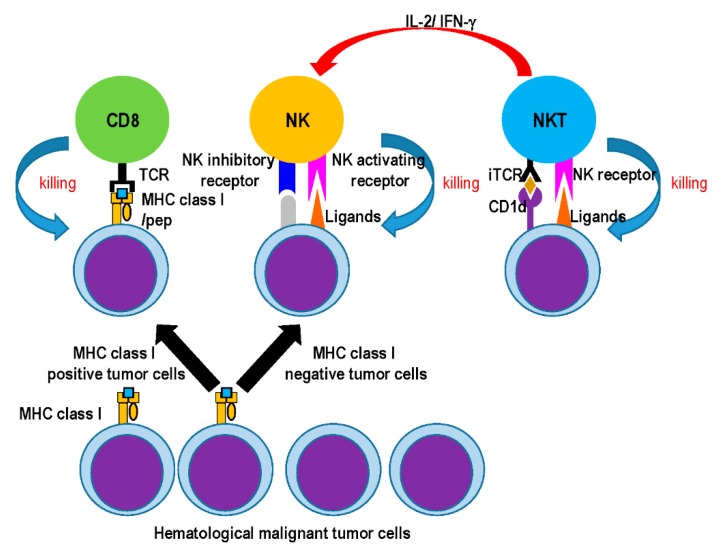
The mode of cytotoxicity effector cells against MHC class I^+^ or MHC Class I^−^ tumor cells. Growing tumor cells comprise MHC class I^+^ and MHC class I^−^ tumor cells. CD8T cells are capable of killing MHC class I^+^ tumor cells (left). On the other hand, NK cells have the potential to kill MHC class I tumor cells (middle). iNKT cells recognize glycolipid/CD1d complex on tumor cells (right). After contact with the ligands, iNKT cells activate NK cells through inflammatory cytokines (IFN-γ and IL-2).

As the effector types of innate lymphocytes, natural killer (NK) cells, natural killer T (NKT) cells, mucosa-associated invariant T (MAIT) cells, and γδ T cells play an important role in immune surveillance against infectious and tumor diseases [4,5]. NK cells are one of the most important populations in the innate immune response and play a pivotal function in cancer immune surveillance. NK cells usually express inhibitory and activating receptors, and they eliminate a variety of abnormal or stressed cells, tumor cells, and infected cells after recognition of target cells [6] (Figure 1). NKT, γδ T, and MAIT cells belong to the family of unconventional T cells. Intriguingly, antigen recognition by these unconventional T cells is not restricted to MHC class I and II molecules [4]. In relation to the anti-tumor response, NKT cells are also well characterized. NKT cells are ordinarily categorized as types I and II [7,8]; type I NKT cells are known as semi-invariant NKT cells (iNKT) as they express a canonical, semi-invariant T cell receptor (TCR), whereas type II NKT cells have a diverse TCR repertoire. Both type I and II NKT cells recognize glycolipid antigens on the CD1d molecule, but their functions in tumor immunity clearly differ [9] (Figure 1). Type I NKT (iNKT) cells are relatively abundant in mice (~1% of T cells), whereas their frequency in humans is low (0.01–0.1% of T cells) [4,7]. γδ T cells lack CD4 and CD8 expression. In human peripheral blood (PB) or lymphoid tissues, 0.5–16% of all CD3^+^ cells is represented by γδ T cells, in which the Vγ9^+^Vδ2^+^ subset is the most dominant in circulation and can respond to small, phosphorylated metabolite antigens [10]. In contrast, the percentage varies between 1% and 4% in mice [4]. MAIT cells belong to another discrete subpopulation of unconventional T cells that are characterized by a limited TCR repertoire. In contrast to NKT cells, MAIT cells are abundant in humans but to a much lesser extent in mice. Most human MAIT cells express an invariant α chain (Vα1.2-Jα33) paired with a limited subset of TCRβ chains (Vβ6 and Vβ2) and recognize vitamin B metabolite antigens presented on the MHC-I related molecule MR1 [4,11]. Such effector-type innate lymphocytes share several common characteristics to some extent, e.g., the secretion of interferon-γ (IFN-γ) and tumor necrosis factor α (TNFα) upon interaction with the ligand or antigen; however, their location, subset, and function differ. Here, we review the role of iNKT and NK cells in the anti-tumor response, particularly in hematological disorders, by highlighting that innate immune cells detect tumors, exert effector functions, and amplify adaptive immune responses. Moreover, we introduce the attempts made to manipulate innate immune responses in cancer.

## 2. iNKT Cell Biology against Cancer

iNKT cells in mice express an invariant TCR formed by the rearrangement of Vα14 and Jα18 TCRα gene segments paired preferentially with Vβ chains including Vβ8.2, Vβ7, or Vβ2 TCRβ gene segments [12,13,14]. On the other hand, iNKT cells in humans express a Vα24-Jα18 rearranged TCRα chain associated with a Vβ11 TCRβ chain [15,16]. Although type II NKT cells are restricted to the CD1d molecule, they have a suppressive role in cancer [9]. Here, we focus on type I NKT cells. 

iNKT cells are capable of producing a large number of cytokines, such as IFN-γ, TNF-α, IL-2, and IL-4, when stimulated by a ligand such as α-galactosylceramide (α-GalCer) [17,18]. Murine iNKT cells can be separated into at least three distinct functional subsets, namely iNKT1, iNKT2, and iNKT17 cells. Differentiation into these three subsets is critically regulated by transcription factors that are relevant to T helper (Th) cells, i.e., T-bet, GATA3, PLZF, and RORγt [19,20]. Development of each type of iNKT cell is generally related to the cytokine milieu encountered upon activation (IFN-γ, IL-4, or IL-17). Their location is also critically regulated. In the peripheral tissues, iNKT1 cells are located in the red pulp of the spleen and liver, whereas iNKT2 cells are located in the T cell zone of the spleen and mesenteric lymph nodes. Although a small population, iNKT17 cells are located in the lung and subcapsular region of the lymph nodes [19,21]. 

Human iNKT cells also develop within the thymus and depend on PLZF expression, similar to murine iNKT cells [22,23]. However, the functional subsets of human iNKT cells are not as well defined as those of mice. In general, these subsets can be divided based on their expression of CD4 and CD8 into CD4^+^ iNKT cells, CD8^+^ iNKT cells, and DN iNKT cells. The DN and CD8^+^ iNKT cells found in humans were reportedly similar to mouse iNKT1 cells, demonstrating increased IFN-γ secretion and cytotoxic function when activated [24,25]. CD4^+^ iNKT cells produce more Th2 type cytokines, such as IL-4 and IL-13, compared with other subsets [24,25]. Human iNKT cells express some NK-related markers such as 2B4, NKG2D, DNAM-1, CD94, and NKG2A, which are dominantly expressed on CD4^−^ iNKT cells [26]. The cytotoxicity of human iNKT cells against target cells may occur via TCR-dependent or -independent signaling.

Anti-tumor surveillance by iNKT cells has been examined in NKT cell-deficient, CD1d-KO, or Jα18-KO mice. For example, inoculation with the carcinogen methylcholanthrene (MCA) led to rapid initiation of spontaneous tumors in Jα18-KO mice [27]. Progressive tumors also developed in prostate TRAMP or KPT pancreatic mouse models using Jα18-KO or CD1d-KO mice, respectively [28,29]. In KPT mice, iNKT cells do not exhibit direct cytotoxicity against tumors but regulate M2-type tumor-associated macrophages (TAMs). In an iNKT cell transfer model, iNKT cells displayed cytotoxicity against TAMs [30]. Another group demonstrated that iNKT cells blocked the function of IL-10-producing neutrophils induced by melanoma tumor cells via serum amyloid A1 (SAA-1) [31]. In humans, the frequency and function of intra-tumor or circulating iNKT cells have been assumed to correlate with overall survival (OS) in several types of cancers [32,33,34,35,36]. However, the frequency of iNKT cells in peripheral blood mononuclear cells (PBMCs) does not always reflect the number of iNKT cells in organs. It is essential to evaluate the functional qualities of iNKT cells and devise a strategy for their reactivation. Thus, iNKT cells play a role in immune surveillance against cancers in a direct or indirect manner.

## 3. NK Cell Biology against Cancer

NK cells, as one of the main populations of innate lymphocytes, play an important role in the defense against infections and cancer. In mice, NK cells are separated into different states of maturation based on their relative expression of CD27 and CD11b. That is, NK cells comprise three subsets: immature NK cells (CD27^+^CD11b^−^), intermediate NK cells (CD27^+^CD11b^+^), and terminal mature NK cells (CD27^−^CD11b^+^) [37]. A subset of the innate lymphoid cell (ILC) family, ILC1, resembles NK cells; however, ILC1 lacks cytotoxic activity [38]. 

Human NK cells are classified by their expression of CD56 and CD16 [6]. CD56^dim^CD16^+^ NK cells are capable of demonstrating a high level of direct cytotoxicity [39], as well as antibody-dependent cellular cytotoxicity (ADCC) using the activating Fc receptor CD16 [40]. On the other hand, CD56^bright^ CD16^−^ NK cells exhibit low cytotoxicity against target cells, but they are capable of producing larger amounts of inflammatory cytokines, such as IFN-γ [41]. CD56^dim^CD16^+^ NK cells can be further subdivided into CD56^dim^CD16^+^CD57^−^NKG2C and CD56^dim^CD16^+^CD57^+^NKG2C^+^ cells. The latter are referred to as adaptive NK cells and possess memory function [42]. 

NK cells possess potent cytolytic activity to kill targeted cells and simultaneously secrete various inflammatory cytokines (IFN-γ, TNF-α, and GM-CSF) and chemokines (CCL3 and CCL5) [6]. The activated status of NK cells depends on the balance between inhibitory and activating signals from receptors. NK cell-activating receptors include NCRs (NKp30, NKp44, and NKp46), c-type lectin (NKG2D), Fc receptor (CD16), and some killer cell immunoglobulin-like receptors (KIRs). On the other hand, NK cell inhibitory receptors include CD94/NKG2A/B, KIR2DL, and KIR3DL [6,43]. These receptor signals are basically triggered by “missing-self” and “induced-self” ligand interactions [44]. The feature of “missing-self” recognition is based on the relative situation where expression of NK-inhibitory MHC-I molecules in the steady state dominates over expression of NK cell-activating ligands, thereby leaving NK cells inactive. In contrast, increased expression of “induced-self ligands” on malignant cells together with reduced MHC-I leads to induction of NK cells possessing potent cytolytic activity. CD56^dim^CD16^+^ NK cells selectively express KIRs, whereas CD56^bright^ CD16^−^ NK cells express CD94/NKG2A [43].

In contrast, tumor cells evade NK cell activity through several strategies. For example, tumor cells and immunosuppressive cells in the tumor microenvironment (TME) produce several factors such as TGF-β and PGE2 to downregulate NKG2D. TGF-β has been shown to downregulate NKG2D on NK cells by decreasing NKG2D transcripts through maturation of miR-1245, which interacts with the 3′-UTR of NKG2D transcripts, leading to repression of NKG2D and decreased DAP10 expression [45]. PGE2 inhibits NKG2D transcription via the adenylate cyclase (AC)/cAMP/protein kinase A (PKA) pathway by binding EP2/4 receptor on the surface of NK cells [46]. Tumor cells (e.g., breast cancer and leukemic cells) reduce or lose the NCR on NK cells via cell–cell contact [47,48,49,50]. PCLP1, a CD34-related sialomucin, expressed on breast cancers decreases the NCR on NK cells via cell–cell contact [50]. Furthermore, tumor cells release soluble forms of NKG2D ligands (e.g., MHC class I chain-related molecules (MIC)A/B and members of the UL16 binding protein (ULBP) family) that inhibit NK cell activation [51]. In fact, MICA/B and ULBPs can often be detected in the sera of patients with various hematopoietic malignancies [51,52,53]. This may block NKG2D on tumor-infiltrating lymphocytes. Similarly, a soluble form of CD155, a ligand for DNAM-1, is often detected in the serum, which is inversely related to prognosis [54]. Circulating BAG6/BAT3, NCR-specific soluble ligands, may inhibit NK cell cytotoxicity by inducing NKp30-specific hyporesponsiveness [55].

## 4. Role of NK and iNKT Cells in Hematological Malignancies (Multiple Myeloma and Leukemia) 

### 4.1. Recent Findings Related to Immunotherapy against Multiple Myeloma and Leukemia

In this review, we focus on the effect of NK and iNKT cells on two hematological malignancies, multiple myeloma (MM) and acute myeloid leukemia (AML). In this section, we first introduce recently developed immunotherapies including T cell-mediated therapies. MM is a B cell malignant disorder characterized by accumulation of a clonal population of transformed plasma cells in the bone marrow [3,4]. Patients with MM are treated with chemotherapy, steroids, immunomodulatory drugs (IMiDs, such as lenalidomide), or proteasome inhibitors (such as bortezomib), in addition to autologous stem cell transplantation (ASCT), which results in improved OS. However, such patients have invariably succumbed to relapse [56]; immunotherapy is, therefore, required. AML is a heterogeneous malignancy based on recurrent genetic and epigenetic abnormalities in hematopoietic stem cells. As a mainstay, these multiple genetic alterations in AML cells lead to the arrest of stem cell differentiation and sometimes accumulation of differentiated myeloblasts in bone marrow. Intensive chemotherapy results in a favorable outcome for AML patients to some extent, but the prognosis of AML is still generally poor, with a 5 year OS of 28% [57]. In particular, the median expected survival of older patients, even those who receive intensive chemotherapy, remains under 1 year [58]. As novel effective therapies, several new targeted agents, e.g., BCL2 inhibitor, hypomethylating agents, and Hedgehog pathway inhibitor, have been clinically examined [59]. In addition, immunotherapy is expected to be useful as a novel therapy for AML.

PD-L1 is highly expressed on myeloma cells, but not on normal plasma cells [60]. Particularly, high PD-L1 expression on myeloma cells was associated with disease progression in patients with monoclonal gammopathy of undetermined significance (MGUS) and asymptomatic MM [61]. Therapeutic efficacy was expected, but monotherapy using anti-PD-1 in MM has not been completely successful. Specifically, there have been some reports of phase I clinical trials. A study involving anti-PD-1 therapy showed no objective responses in 27 patients with relapsed or refractory MM (RRMM) [62]. A similar study with anti-PD-1 monotherapy for RRMM demonstrated stable disease in 57% of patients [63] (Table S1). Lenalidomide downregulates PD-1 expression of myeloma patient-derived T cells, allowing the restoration of their cytotoxicity. However, combination anti-PD-1 and IMiD therapy has been discontinued due to increased risk of patient death in addition to an absence of significant difference in the phase III clinical trial (KEYNOTE183/NCT02576977, KEYNOTE185/NCT02579863) [64,65] (Table S1). Lenalidomide has been shown to disrupt the cross-talk between myeloma and stromal cells in the tumor microenvironment. Through this interaction, the secretion of pro-angiogenic and anti-inflammatory molecules was decreased, whereas PD-1 and PD-L1 expression in MM cells was either downregulated or upregulated [66,67,68]. There are a couple reports that treatment with hypomethylating agents (HMA) resulted in a dose-dependent upregulation of checkpoint molecules including PD-1, PD-L1, and CTLA4 in human and murine myeloid malignancies [69,70] Patients with relapsed/refractory AML were treated with nivolumab and azacytidine (HMA) in a phase II study. The overall response rate (ORR) was 33%, including 22% patients with complete remission and 10% with hematologic improvements (HI) that were maintained for >6 months [71] (Table S2). Due to an encouraging response from combination HMA and anti-PD-1 therapy, phase II trials of ICB combination therapies have been initiated (NCT03092674 and NCT02775903).

Bispecific T cell engagers (BiTEs) are a form of scFv-based antibody, comprising only the variable heavy and light chain regions. BiTEs target not only a tumor epitope but also a T cell antigen, usually CD3. By T cell-tumor cell adherence mediated via BiTEs, T cells are activated and exhibit cytotoxicity against tumor cells. Since BiTEs are independent of TCR and MHC expression, they offer several potential advantages over monospecific antibodies. B cell maturation antigen (BCMA) has been the focus of a majority of BiTEs to date. In a first-in-human study of AMG 420 immunotherapy (NCT02514239), which binds to BCMA on MM cells and CD3 on T cells, 42 patients received AMG 420 (0.2–800 μg/d) (Table S1). There were 13/42 responders (6 stringent complete responses (sCRs), 3 complete responses (CRs), 2 very good partial responses (VGPRs), and 2 partial responses (PRs)) [72]. A number of BiTEs targeting CD33, CD123, and CLEC12A/CCL-1 (CD371) have been developed for AML [73,74,75,76]. The full-length human IgG1 bispecific antibody MCLA-117 targeting CLEC12AxCD3 is also currently being tested in AML (NCR03038230) [77]. Flotetuzumab (a bispecific antibody that targets both CD3 and CD123) demonstrated measurable anti-leukemic effects in 8/14 patients with chemotherapy-refractory AML (57%), and two of these patients achieved complete remission [75]. As an alternative to BiTEs, NK-targeting bi- (BiKEs) and tri-specific killer engagers (TriKEs) have been developed [78]. These are composed of small molecules containing the variable portion (V_H_ and V_L_) of an antibody linked to one (BiKE) or two (TriKE) variable portions from other antibodies of different specificity. As the CD56^dim^CD16^hi^ NK subset has a high cytotoxic capacity, agonistic anti-CD16 scFv component is used for targeting NK cells. A phase I/II clinical trial of GTB-3550 (CD16/IL-15/CD33) TriKE for high-risk hematological malignancies is currently underway (NCT03214666). Recently, another new approach, natural killer cell engagers (NKCEs), has been reported. NKCEs are designed to target two activating receptors, NKp46 and CD16, on NK cells in addition to tumor antigen on cancer cells. As NKp46 is expressed on NK cells in the tumor site, the co-targeting of NKp46 and CD16 may be prospective [79]. 

Major efforts have been made to develop the adoptive transfer of T cells expressing genetically engineered chimeric antigen receptors (CAR T cells) in hematologic malignancies. An scFv-based CAR directed against BCMA (CART-BCMA) was tested in a phase I clinical trial that included patients with relapsed MM, where it showed a toxicity profile similar to that of CD19-specific CAR T cells with excellent clinical response rates ranging from 20% to 64% [80] (Table S1). CAR T cell therapy is quite promising in the presence of well-defined lineage-specific antigens such as CD19 and BCMA in B cell malignancies. However, one of the major challenges in adopting CAR T cell therapy toward AML is the lack of a proper antigen in AML, and only a few clinical trials have been conducted so far. For example, a phase I study examined autologous CAR anti-Lewis Y (LeY) T cell therapy in AML [81]. The infused CAR T cell therapy resulted in a good response in three patients, and cytogenetic remission was recorded in one patient without severe cytotoxicity. Recently, NK cells have been the focus of CAR-expressing cell research because they can be used in an allogeneic setting without causing graft-versus-host disease. Clinical trials involving immunotherapies using several types of CAR NK cells have been initiated (e.g., NCT03415100, NCT03056339, and NCT03692767) [82]. In addition, tumor-specific TCR-transduced T cells (TCR-T) for hematological malignancies have been developed and tested in clinical trials. Adoptive transfer of Wilms’s tumor 1 (WT1)-specific TCR-T has been tested in patients with refractory AML, high-risk MDS [83], and AML after allogeneic hematopoietic cell transplantation [84] (Table S2). Adoptive transfer of NY-ESO-1-specific TCR-T has been tested in MM after ASCT [85] (Table S1). The results suggest that this strategy may be promising for maintenance of stable disease or prevention of disease recurrence. 

### 4.2. Role of iNKT Cells against Multiple Myeloma and Leukemia

Sialylated glycolipids may become targets for NKT cells. Sialic acid-containing glycolipids on tumor cell membranes including lacto- and neolacto-series glycolipids and globosides (glycosphingolipid (GSL)-containing acetylated amino sugars and simple hexoses) can be modified in tumor progression. Several types of tumors, e.g., melanoma, small-cell lung cancer (SCLC), sarcoma, and neuroblastoma, highly express gangliosides GD3, GM2, GM3, and GD2 in comparison with corresponding normal tissue [86,87,88,89]. Particularly, GD3 expressed on melanoma is known to activate both CD4^+^CD8^−^ NKT and CD4^−^CD8^−^ NKT cells to produce IL-4 [90], whereas CD4^−^ CD8^−^ NKT cells also react with GM3 as a tumor-associated suppressive glycolipid in a CD1d-restricted manner [91]. On the other hand, GD3 isolated from the polar lipid fraction of ovarian cancer-associated ascites is an inhibitory factor that prevents NKT cell activation [92]. HPLC analysis of CD138^+^ plasma cells from patients with MM revealed that GM3 and GM2 are the predominant GSLs [93], but Park et al. reported that NKT cells do not react with GM2 [91]. This indicates that some tumor-derived gangliosides act as NKT cell ligands and might be related to prognosis in some cancers, but not all malignancies. Primary leukemic cells from patients with AML, especially the M1 subtype, often express GSLs, such as lactotriaosylceramide, GM3, and neolactotetraosylceramide [94]. GM3 inhibits IL-4, but not IFN-γ, production of iNKT cells in response to α-GalCer [91]. It is also reported that peroxisome-derived lipids (PDLs), 1-*O*-1′-(Z)-hexadecenyl-2-hydroxy-*sn*-glycero-3-phosphoethanolamine, and 1-*O*-1′, 9′-(Z,Z)-octadecadienyl-2-hydroxy-*sn*-glycero-3-phosphoethanolamine are potential ligands of iNKT cells in the mouse thymus [95]. Xu et al. reported that the relative quantity of PDLs extracted from the bone marrow of human patients with AML and acute lymphoblastic leukemia (ALL) was higher than that extracted from healthy donors [96]. 

Vα24^+^ iNKT cells can apparently be detected in PBMCs and bone marrow mononuclear cells (BMMNCs) in patients with myeloma. However, Vα24^+^ iNKT cell frequency and disease progression are inversely correlated [97,98,99,100] due to at least the expression of CD1d on primary myeloma cells [98]. Premalignant and early-stage myeloma cells show high expression of CD1d, which decreases in the advanced stages [101]. In addition, IFN-γ production from primary iNKT cells in PBMCs is decreased in progressive MM compared to MGUS and asymptomatic MM. Despite a severe reduction in numbers, iNKT cell populations can be expanded by α-GalCer-loaded CD1d^+^ cells/Gal (CD1d/Gal) leading to increased production of IFN-γ. The restoration suggests that iNKT cells may be in an anergic state rather than a state of impairment. iNKT cell frequency in patients with MM treated with steroid was low. However, iNKT cell populations expanded and produced IFN-γ by CD1d/Gal stimulation even in steroid-treated patients, indicating that IFN-γ-producing iNKT cells in BM are preserved in most patients with MM [99]. When patients with MM were injected with α-GalCer-pulsed dendritic cells (DCs) (DC/Gal), the iNKT cell pool expanded 100-fold and lasted for several months [102]. In patients administered DC/Gal plus lenalidomide, iNKT cell populations expanded transiently and declined thereafter. Despite this, the clear activation of NK cells with upregulation of NKG2D was shown. Furthermore, there was a decline in M spike derived from myeloma cells [103]. ASCT is one of the standard therapies in MM. The frequency and absolute numbers of iNKT cells were greatly decreased but did not recover to the pre-ASCT level during lenalidomide maintenance therapy [104].

A few studies have been conducted on the immunotherapeutic potential of iNKT cells in myeloid leukemia. Purified iNKT cells from primary PBMCs were shown to be α-GalCer/CD1d reactive. Primary leukemic cells from AML, especially M4 and M5 types or juvenile myelomonocytic leukemia, express CD1d. Seven days after immobilized α-GalCer/CD1d-tetramer restimulation, iNKT cells exhibited cytotoxicity against autologous leukemic cells and produced IFN-γ, TNF-α, IL-2, and IL-4 upon stimulation with α-GalCer-pulsed leukemia cells [105]. Another report demonstrated that an expanded iNKT and NK cell mixture from the PBMCs of patients with AML displayed anti-leukemic lytic activity in vitro [106].

### 4.3. Role of NK Cells in Protection against Multiple Myeloma and Leukemia 

NK cells are considered key effector cells against myeloma cells in that they can recognize and kill myeloma cells sufficiently via their NK receptors, especially NKG2D, DNAM-1, and NKp30. However, NK cell activity in advanced MM is sometimes significantly impaired. NKG2D and DNAM-1_expression on NK cells is downregulated in correlation with disease progression [107,108], which might be because of the release of soluble ligands, cytokines, or exosomes from myeloma cells [109,110]. Myeloma and T regulatory (Treg) cells from patients with MM secrete high levels of TGF-β [111]. Since Treg cells from breast cancer tissues have been shown to downregulate NK-activating receptors and inhibit NK cytotoxicity [112], this might also be true for MM. Owing to the expression of PD-1 on NK cells and PD-L1 on myeloma cells [113], PD-1/PD-L1 interactions promote NK cell functional exhaustion. This phenomenon could be potentially reversible by ICB treatment. 

Lenalidomide not only acts as a direct anticancer drug but also belongs to the class of immunomodulatory drugs. It increases co-stimulatory receptor expression on NK cells, as CD16 and lymphocyte function-associated antigen-1 (LFA-1) lower the threshold for NK cell activation [114,115]. Intriguingly, the interaction between NK cells and lenalidomide is controversial. Lenalidomide decreases activating receptor expression on NK cells and affects the secretion of IFN-γ by NK cells [116]. Lenalidomide-stimulated NK cells in patients with chronic lymphocytic leukemia (CLL) display reinforced cytotoxic activity and NK cell-mediated tumor surveillance [117,118,119].

In leukemia, the anti-leukemic activity of NK cells inversely correlates with the progression of AML. NK cell function is suppressed at diagnosis and relapse but can be restored at complete remission (CR) [120,121]. Furthermore, an increased proportion of mature NK cells is significantly correlated with long-term imatinib-free remission in chronic myeloid leukemia (CML) [122]. In fact, NK cells are capable of killing primary leukemic cells from patients with CML, AML, or MDS [123,124]. The molecular specificity of NK cell-mediated cytotoxicity of leukemic cells is based on several receptor–ligand interactions. For instance, NKG2D and DNAM-1 receptors and NCRs (NKp30 and NKp46) are reportedly important for targeting AML and CML blasts [125]. Indeed, expression of ULBP1 as an NKG2D ligand on blasts is significantly associated with improved 2 year overall and relapse-free survival [126].

## 5. Therapeutic Efficacy of iNKT and NK Cell Potentials against Multiple Myeloma and Leukemia

### 5.1. iNKT and NK Cell-Mediated Immunotherapy

Missing-self recognition by NK cells plays a major role in the elimination of tumor cells in the allogeneic setting. MM cells ordinarily express high levels of HLA-class I on the cell surface [127,128,129]. Considering that KIR ligand-mismatched NK cells are active effector cells, infusion of KIR ligand-mismatched NK cell donors could be an effective approach against MM cells [128]. These cells may still be effective even if the tumor microenvironment is in poor condition, i.e., exhibiting hypoxia and producing high levels of PGE2 and lactate [130]. In addition, ex vivo expanded clinical NK cell products harbor a high percentage of NKG2A^+^ NK cells. However, HLA-E expression on primary MM cells is not sufficient to trigger potent inhibitory signaling via CD94^−^NKG2A [131]. Creation of missing-self based on interference with NKG2A would potentiate activation of KIR ligand-mismatched NK cells against tumors expressing high levels of HLA-E. KIR ligand interaction using an anti-HLA antibody can be blocked to create missing-self with monoclonal anti-KIR antibody, resulting in enhanced killing of primary MM tumor cells by haploidentical KIR ligand-mismatched NK cells [132]. Phase I or II clinical studies of anti-KIR antibody (IPH2101) in patients with relapsed/refractory or smoldering MM demonstrated that despite being safe and well tolerated, anti-KIR antibody did not bring about therapeutic benefit [133,134]. To unravel the unexpected lack of clinical response, a follow-up study using an injection of IPH2101 demonstrated reduced KIR2D expression on the surface of NK cells as well as reduced function of NK cells [135]. That is, KIR2D removal of anti-KIR-treated NK cells was caused by trogocytosis; monocytes removed antibody-bound molecules from the cell surface. Blocking KIR by anti-KIR antibodies could result in uneducated, hyporesponsive NK cells and, subsequently, limited antibody effectiveness in vivo. Despite the single drug employed, combination therapy using anti-KIR antibody and lenalidomide enhanced NK cell function, resulting in objective responses [136].

The ADCC of myeloma cells by NK cells through CD16 has been employed widely. The response relies heavily on co-stimulation with activating receptors, i.e., NKG2D, DNAM-1, and NCRs on NK cells, along with their respective ligands on myeloma cells. The CD38 antibody daratumumab demonstrated marked cytotoxicity against myeloma cells through ADCC, which relies on binding of NK cell CD16 (FcγRIIIA), complement-dependent cytotoxicity (CDC), and antibody-dependent cellular phagocytosis (ADCP). Daratumumab displayed clinical relevance in combination with IMiDs (lenalidomide and pomalidomide) [137]. Phase III trials using daratumumab with either lenalidomide/dexamethasone (POLLUX) or bortezomib/dexamethasone (CASTOR) have shown impressive responses in RRMM with a 12 month PFS of 83.2% (daratumumab group) versus 60.1% (control group) for standard care, and 60.7% (daratumumab group) versus 26.9% (control group), respectively [138,139] (Table S1). All-trans retinoic acid upregulated CD38 expression and reduced expression of complement inhibitors CD55 and CD59 on MM cells, leading to markedly improved daratumumab-mediated ADCC and CDC [140]. Elotuzumab is a humanized anti-SLAMF7 (signaling-lymphocyte-activating molecule F7) monoclonal antibody that targets SLAMF7 on myeloma cells and can lead ADCC via CD16 on NK cells and ADCP by macrophages [141]. Since SLAMF7 is also expressed on NK cells, the cross-linking of SLAMF7 on NK cells by a treatment with elotuzumab induces NK cell activation via immunoreceptor tyrosine-based switch motifs (ITSM)-mediated signaling. These ITSM-mediated signaling enhances calcium signaling from ITAM-linked activating receptor, through an interaction between NKp46 and NKG2D and their ligands expressed on myeloma cells [142] or stimulates macrophage-mediated ADCP [143]. The phase III ELOQUENT-2 study (elotuzumab plus lenalidomide and dexamethasone vs lenalidomide and dexamethasone) reported a 29% reduction in risk of death or disease progression at 4 years in the elotuzumab arm of the trial [144] (Table S1).

One approach is to activate allogeneic NK cell populations in vitro and transfer them along with HSCT into patients with AML [145,146,147]. In fact, infusion of allogeneic NK cells was performed in high-risk patients without HSCT—38% of whom achieved complete or partial remission [148], suggesting the susceptibility of myeloid blasts to NK cell infusions. In patients with AML after chemotherapy, infusions of activated memory-like NK cells from allogeneic donors were well tolerated with no graft-versus-host disease nor any other serious adverse events. The ORR, including CR with incomplete blood count recovery, was 55% (5 of 9) [149] (Table S2).

The iNKT-mediated NK activation response is well known. As an important factor for iNKT and NK cell activation in iNKT cell ligands (such as α-GalCer), IFN-γ production by iNKT cells and, subsequently, by NK cells is critical in tumor protection [150]. To demonstrate the contribution of iNKT cells in this anti-metastatic effect, adoptive transfer of iNKT cells into Jα281^−/−^ mice that received α-GalCer and B6 WT NK1.1^+^ T cells exhibited complete protection from B16F10 tumors. Comparatively, transfer of NK1.1^+^ T cells alone (vehicle-treated) was insufficient to confer protection. In general, CD1d^−^ tumor cell lines are resistant to iNKT cells, whereas CD1d^+^ cell lines, such as Jurkat and U937 cell lines, are susceptible [151]. iNKT cells can mediate potent anti-tumor activity directly by targeting CD1d, as well as indirectly by activating NK cells. NK cell cytotoxicity mostly depends on NK cell activation by IL-2 and IFN-γ production from TCR-stimulated iNKT cells [151] (Figure 1). As an anti-myeloma effect, we and others have demonstrated that NK cell cytotoxicity against primary myeloma cells depends on expression of NKG2D and DNAM-1, as well as perforin/granzyme B [99]. In fact, such characteristics of NK cells were shown in DC/Gal-treated patients [103]. 

### 5.2. Next-Generation iNKT Cell-Mediated Immunotherapy against Hematological Malignancies 

Next-generation therapeutic strategies against hematologic malignancies have been explored. As previously described, iNKT cell therapy by autologous DC/Gal administration was used. To develop a framework for iNKT-mediated immunotherapy, we initially demonstrated that co-administration of OVA protein antigen with iNKT ligand resulted in the generation of antigen-specific CD8^+^ T cells in a murine model [152,153]. To develop an efficient therapy, we proposed using an all-in-one cell type that expressed tumor antigen as well as CD1d and was simultaneously loaded with the iNKT cell ligand α-GalCer, a cell that we reported as an adjuvant vector cell [154,155]. We first showed conclusively that CD1d^+^ tumor/Gal in hematological malignancy models, including a lymphoma (EL4), leukemia (WEHI3B), and plasmacytoma (J558) model, can induce antigen-specific CD4^+^ and CD8^+^ T cell immunity [154,155]. Another group also reported results from α-GalCer-loaded B cell lymphoma (Eμ-myc tumor) combined with an agonistic antibody targeting 4-1BB (CD137) [156]. These studies demonstrated antigen-specific effector T cell-mediated survival. These tumor/Gal vaccines would be useful in an autologous setting for hematological malignancies. To further develop this approach, we established an artificial adjuvant vector cell (aAVC) as a novel putative type of cancer vaccine platform that incorporates in vivo iNKT-licensed DC therapy [157]. These cells express the α-GalCer-CD1d complex on their surface and tumor antigen proteins (e.g., MART-1 and Trp2) inside the cells. The aAVCs initially activate iNKT cells directly and NK cells are soon activated by iNKT cells producing IFN-γ. In turn, these innate killer iNKT/NK cells reject the adjuvant vector cells, and the dying adjuvant vector cells are then taken up by DCs in situ. DCs in the lung, liver, and spleen undergo maturation by their interaction with iNKT cells. Finally, the mature DCs in situ can generate antigen-specific CD4^+^ and CD8^+^T cells [7,158,159]. As evidence of anti-tumor activity, particularly against hematological malignancies, we demonstrated that after treatment with WT1-expressing aAVC (aAVC-WT1), J558-WT1 myeloma cell-bearing mice survived for more than 6 months, and the establishment of relevant myeloma cells but not irrelevant WT1-defected tumors was blocked when rechallenged with the same tumor. Thus, aAVC therapy has the potential to induce memory T cells [7,159,160]. Currently, we are conducting a phase I trial for refractory and relapsed AML. 

## 6. Conclusions

In this review, we summarized the importance of the biology of NK and iNKT cells, particularly in MM and AML. Although NK cells are classified by maturation status and function, they are regulated by the balance between inhibitory and activating signals from the receptors. Recent findings demonstrate that one NK cell population possesses potent cytolytic activity against targeted cancer cells, while others uniquely secrete various inflammatory cytokines (IFN-γ, TNF-α, and GM-CSF) and chemokines (CCL3 and CCL5) [6]. Of note, iNKT cells are known to interact with NK cells. Several preclinical and clinical studies indicated that IFN-γ production by iNKT cells may serve as a biomarker for anti-tumor immunity in mouse models and cancer patients. We particularly focused on the clinical findings in MM and AML, and discussed the relationship between disease progression, therapy, and immunology. Interestingly, PD-L1 expression on myeloma cells or NK and iNKT cell function in PBMCs or BMMNCs was associated with disease progression in patients with MGUS and asymptomatic or advanced-staged MM. However, iNKT cell function in advanced-stage cancer patients can be converted by α-GalCer-loaded CD1d^+^ cell therapy.

In the latter part of this review, we discussed therapies and the relationship between drug and NK and iNKT cell responses. The efficacy of antibody therapy with elotuzumab and daratumumab at least partly depends on NK cells. In addition, iNKT activation can directly enhance DC-mediated reactions, resulting in T cell immunity. Therefore, we discussed iNKT cell-triggering DC therapy as next-generation immunotherapy. Thus, NK and iNKT cells are stimulated or sometimes regulated by immune-modulating drugs (IMiDs, or anti-PD-1 antibody), cytokines, and chemokines, and they can, subsequently, control other immune cells. These findings provide the possibility to use the combination therapy using different types of drugs. Further studies are warranted to establish new immunotherapies.

## Supplementary Materials

The following are available online at https://www.mdpi.com/2072-6694/12/4/817/s1, Table S1: Selected clinical trials of the immunotherapies for MM, Table S2: Selected clinical trials of the immunotherapies for AML.
